# Prurigo of the Genital and Anal Regions

**Published:** 1852-02

**Authors:** 


### PATHOLOGY AND PRACTICE OF MEDICINE.

*Prurigo of the Genital and Anal Regions.*—Various remedies are from time to time recommendeded in the journals for this disagreeable and obstinate affection. The London Lancet, for December 15th, gives an application of M. Tournié's, as having been used with much success. "The affected spot is to be rubbed twice a day with calomel ointment, (one to two drachms of calomel to ounce of axunge), and, after each application, dredged with a powder, consisting of four parts of starch to one of powdered camphor." We have used a great variety of applications, for the relief of this unmanageable affection, and have derived most advantage from a cerate made of calomel (ʒi), in Goulard's cerate (℥i.)

